# Complete Genome Sequences of Two Escherichia Phages Isolated from Wastewater in Finland

**DOI:** 10.1128/genomeA.00401-18

**Published:** 2018-05-31

**Authors:** Saija Kiljunen, Anu Wicklund, Mikael Skurnik

**Affiliations:** a Department of Bacteriology and Immunology, Medicum, Research Programs Unit, Immunobiology Research Program, University of Helsinki, Helsinki, Finland; b Division of Clinical Microbiology, HUSLAB, University of Helsinki and Helsinki University Hospital, Helsinki, Finland

## Abstract

Escherichia phages vB_EcoM-fFiEco06 and vB_EcoM-fHoEco02 were found to have 167,076-bp and 167,064-bp genomes, respectively. They are members of genus T4virus, and they are 99.96% identical to each other. The host ranges of the phages are different, probably due to a few differences in their tail protein amino acid sequences.

## GENOME ANNOUNCEMENT

Phage vB_EcoM-fFiEco06 (fFiEco06) was isolated from a mixture of municipal wastewater samples collected from several towns in Finland using clinical Escherichia coli strain 123738 (obtained from HUSLAB, Helsinki, Finland) as a host. Phage vB_EcoM-fHoEco02 (fHoEco02) was isolated from a hospital wastewater sample collected in Helsinki, Finland, using the same host. Both phages form clear ∼2-mm-diameter plaques and show the morphology of a typical member of the Myoviridae when examined with transmission electron microscopy. The phages had rather narrow host ranges. Out of 200 clinical E. coli strains tested, fFiEco06 infected 9 strains and fHoEco02 infected 17 strains, indicating that even though the two phages were isolated using the same host, their host specificities are slightly different.

Phage DNA was isolated with the Invisorb Spin virus DNA minikit (Stratec Biomedical). Next-generation sequencing was performed at the Institute for Molecular Medicine Finland (FIMM) Technology Centre Sequencing Unit. DNA libraries were constructed with the Nextera DNA sample preparation kit, and paired-end sequencing was done using a MiSeq PE300 sequencer (Illumina, San Diego, CA, USA) with a 300-nucleotide (nt) read length. Reads were assembled with the A5-miseq integrated pipeline for *de novo* assembly of microbial genomes (1). The pipeline yielded 167,076-bp and 167,064-bp contigs for fFiEco06 and fHoEco02, respectively, with 271- and 145-fold median coverage depths. The G+C content of both genomes was 35.3%.

Phage genomes were aligned pairwise to each other and to the enterobacterial phage T4 sequence (GenBank accession number NC_000866), the type virus of genus T4virus in viral family Myoviridae and subfamily Tevenvirinae, with EMBOSS Stretcher (2). fFiEco06 and fHoEco02 were 99.96% identical to each other, and they showed 82.17% and 82.32% identity to T4, respectively. The two phages can thus be considered representatives of the same species and as members of T4virus. The genomes were annotated using Rapid Annotations using Subsystems Technology (RAST) (3–5), BLASTP (6), and tRNAscan-SE version 2.0 (7). fFiEco06 and fHoEco02 had 274 and 272 protein-coding genes, respectively, and both phages had 10 tRNA genes. The difference in the number of protein-coding sequences (CDSs) between the two phages results from small indels in the fHoEco02 genome that cause premature stop of CDSs corresponding to fFiEco06 FE6_028 and FE6_212, coding for small outer capsid (Soc) protein and a hypothetical protein, respectively. In addition to these differences, there is a 9-bp deletion in the fHoEco02 genome just before the start codon of HE2_107, coding for the rI.1 conserved hypothetical protein, which may influence its translation.

Even though the sequences of fFiEco06 and fHoEco02 were almost identical, the host specificities of the two phages were different. Therefore, we made pairwise comparisons of the amino acid sequences of their tail proteins with EMBOSS Stretcher. There were 1- to 2-amino-acid differences between CDSs coding for tail sheath monomers (FE6_175 and HE2_174), long tail fiber proximal subunits (FE6_253 and HE2_251), long tail fiber distal subunits (FE6_256 and HE2_254), and tail fiber adhesins (FE6_257 and HE2_255), which probably explain the different host ranges of the phages.

### Accession number(s).

The genomic sequences of vB_EcoM-fFiEco06 and vB_EcoM-fHoEco02 have been deposited in GenBank under the accession numbers MG781190 and MG781191, respectively.
